# Diabetes Mellitus in Acute Coronary Syndrome

**DOI:** 10.3390/life13112226

**Published:** 2023-11-19

**Authors:** Panagiota K. Stampouloglou, Artemis Anastasiou, Evanthia Bletsa, Stavroula Lygkoni, Flora Chouzouri, Maria Xenou, Ourania Katsarou, Panagiotis Theofilis, Konstantinos Zisimos, Dimitris Tousoulis, Manolis Vavuranakis, Gerasimos Siasos, Evangelos Oikonomou

**Affiliations:** 1 3rd Department of Cardiology, Thoracic Diseases General Hospital “Sotiria”, National and Kapodistrian University of Athens, 11527 Athens, Greece; stampoulogloupanagiota@gmail.com (P.K.S.); artemisan93@gmail.com (A.A.); evabletsa@gmail.com (E.B.); vinagoni@hotmail.com (S.L.); chouzouriflora@gmail.com (F.C.); xenomary@gmail.com (M.X.); zisimoskostas@gmail.com (K.Z.); vavouran@otenet.gr (M.V.); ger_sias@hotmail.com (G.S.); 2 1st Department of Cardiology, “Hippokration” General Hospital, National and Kapodistrian University of Athens, 11527 Athens, Greece; panos.theofilis@hotmail.com (P.T.); drtousoulis@hotmail.com (D.T.)

**Keywords:** diabetes mellitus, biomarker, acute coronary syndrome, coronary artery disease

## Abstract

The global prevalence of diabetes mellitus (DM) has led to a pandemic, with significant microvascular and macrovascular complications including coronary artery disease (CAD), which worsen clinical outcomes and cardiovascular prognosis. Patients with both acute coronary syndrome (ACS) and DM have worse prognosis and several pathophysiologic mechanisms have been implicated including, insulin resistance, hyperglycemia, endothelial dysfunction, platelet activation and aggregations as well as plaque characteristics and extent of coronary lesions. Therefore, regarding reperfusion strategies in the more complex anatomies coronary artery bypass surgery may be the preferred therapeutic strategy over percutaneous coronary intervention while both hyperglycemia and hypoglycemia should be avoided with closed monitoring of glycemic status during the acute phase of myocardial infraction. However, the best treatment strategy remains undefined. Non-insulin therapies, due to the low risk of hypoglycemia concurrently with the multifactorial CV protective effects, may be proved to be the best treatment option in the future. Nevertheless, evidence for the beneficial effects of glucagon like peptide-1 receptor agonists, dipeptidyl-peptidase 4 inhibitors and sodium glycose cotransporter 2 inhibitors, despite accumulating, is not robust and future randomized control trials may provide more definitive data.

## 1. Introduction

It has been well established over the last decades that the rising prevalence of diabetes mellitus (DM) worldwide has resulted in a pandemic, magnifying its impact given that only in 2019 the number of deaths attributed to the disease has been estimated to be around 2 million [1].

Considering the microvascular, along with the macrovascular, complications of the disease, DM is linked with comorbidities such as cerebrovascular disease, peripheral vascular disease, chronic kidney disease, and, last but not least, coronary artery disease (CAD), adversely affecting the clinical outcomes and cardiovascular (CV) prognosis [2]. In fact, approximately 25–30% of the subjects admitted with acute coronary syndrome (ACS) in hospitals have DM. Furthermore, patients with ACS and DM, compared to individuals with ACS but with no DM, present poorer outcomes regarding CV morbidity and mortality [3].

Moreover, recent studies indicate that patients with ACS and type 2 DM (T2DM) have prolonged hospitalization, higher 30-day readmission rates, as well as worse mortality rates compared to the general population, due to specific pathophysiological mechanisms implicating metabolic pathways [4,5]. So far, to interpret these correlations, it has been determined that patients with DM have a greater burden of atherogenic risk factors such as hypertension, obesity, and dyslipidemia when compared to patients without. At the same time, rough estimations also show that 70–75% of all patients with established CAD present, at least to some degree, disruption of the normal glucose homeostasis. This observation even led to the recent recommendation for screening these patients for potential T2DM when establishing the presence of a CV disease, highlighting the bidirectional effect of T2DM on ACS and vice versa [1,6,7]. Therefore, in this article we review the pathophysiologic link between DM and ACS as well as the current data on the diagnosis and explicit management of the linked entities.

## 2. Pathophysiology of ACS in Patients with DM

Pathophysiological disturbances in patients with DM experiencing an ACS can be classified in four main categories: (1) insulin resistance, (2) endothelial dysfunction, (3) plaque alteration, (4) platelet activation and coagulopathy. These disturbances trigger signaling factors that influence endothelial cells, vascular smooth muscle cell (VSMCs), macrophages, as well as platelets [8]. The interplay between these molecular factors ultimately provokes a highly atherosclerotic, pro-inflammatory, and prothrombotic state [9]. Finally, all these factors have an adverse impact on both microcirculation and epicardial coronary arteries [10].

Hyperglycemia and insulin resistance are the hallmarks of DM. Insulin resistance in skeletal muscle leads to a decrease in glucose disposal, compensatory hyperinsulinemia, and the use of free fatty acids (FFAs) as an energy source. Considering that myocardial glucose uptake is disrupted, myocardium uses FFAs instead of glucose, leading to further hypoxia [11]. In addition, DM is characterized by vascular endothelial dysfunction and accelerated atherosclerosis. The endothelium, consisting of squamous cells adjacent to basal lamina, does not only serve as a boundary between vessel lumen and wall but also as an endocrine organ regulating the vessel homeostasis, including vasoconstriction and relaxation [12]. Endothelial dysfunction appears quite early before the onset of coronary artery disease (CAD), and even before the clinical diagnosis of DM [13]. The imbalance between vasodilatation and vasoconstriction, in combination with a highly prothrombotic, inflammatory, and oxidative state, severely impairs endothelial function [14].

**Figure 1:**
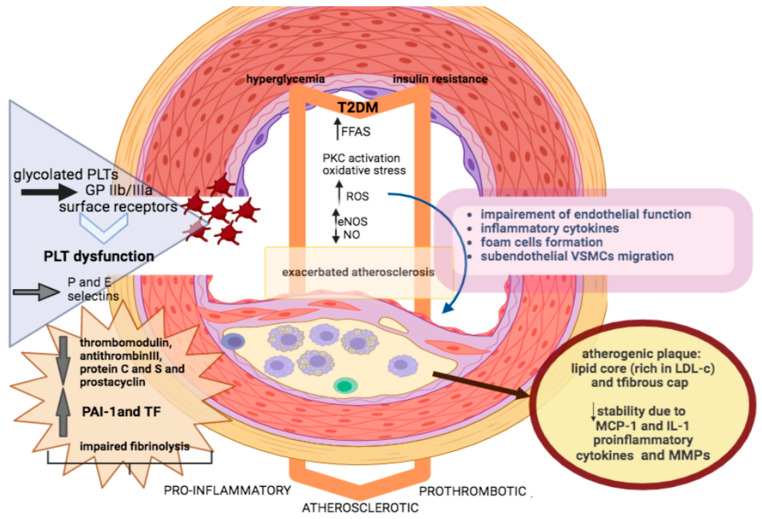
Endothelial pathophysiology in diabetes. T2DM—type 2 diabetes mellitus; FFAs—free fatty acids; PKC—protein kinase; eNOS—endothelial nitric oxide synthase; ROS—reactive oxygen species; NO—nitric oxide; VSMCs—vascular smooth muscle cells; PLT—platelet; GP IIb/IIIa—glycoprotein IIb/IIIa; PAI-1—plasminogen activator inhibitor 1; LDL-c—low-density lipoprotein cholesterol; MCP-1—monocytechemoatrractant; IL-1—interleukin 1; MMPs—matrix metalloproteinases; ↓—Decrease; ↑—Increase.

Hyperglycemia and FFAs activate protein kinase C (PKC) and induce impaired activity of endothelial nitric oxide synthase (eNOS), thus impeding the production of nitric oxide (NO) and thus endothelium-dependent vasodilation [15] (Figure 1). Meanwhile, hyperglycemia induces secretion of reactive oxygen species (ROS) and inhibits the activity of eNOS and other compensatory vasodilators, such as prostacyclin [16]. ROS in the arterial wall do not only directly impair endothelial function, but at the same time promote the expression of inflammatory cytokines into a vicious cycle causing further oxidative damage [17]. ROS also contribute to foam cell formation and sub-endothelial migration of VSMCs [18]. Additionally, increased production of vasoconstrictive factors, such as endothelin-1 (ET-1) and angiotensin II has been reported among patients with DM. The imbalance in vasoconstriction and vasodilatation leads to increased endothelial permeability and the secretion of cytokines, promoting a proinflammatory state [19].

DM exacerbates the development and progression of atherosclerosis. VSMCs proliferation, macrophage infiltration, and foam cell formation result in the development and progression of atheromatic plaque. Of note, atherosclerotic plaque is characterized by a lipid core and a fibrous cap. Small dense particles with increased triglycerides or low-density lipoprotein cholesterol (LDL-c) are more atherogenic, accelerating the atherosclerotic process [20]. Moreover, the inflammatory substrate of DM is a key determinant of endothelial impairment, resulting in the expression of adhesion molecules on the endothelial surface and sub-endothelial accumulation of inflammatory cells. The activity of inflammatory lymphocytic infiltration in atherosclerotic plaque is remarkable in patients with DM [21]. Proinflammatory cytokines such as monocyte chemoattractant protein-1 (MCP-1) and intereleukin-1 (IL-1) contribute to reduced stability of atherosclerotic plaque. Higher levels of matrix metalloproteinases among patients with DM contribute to increased collagen breakdown and decreased synthesis in the fibrous cap of atherosclerotic plaque, subsequently resulting in plaque rupture and thrombus formation, clinically manifesting as ACS [22]. Therefore, patients with DM are not only predisposed to plaque formation but are also at higher risk of plaque rupture.

Interestingly, endothelial dysfunction is associated with platelet dysfunction and impaired fibrinolysis [23]. Platelets under hyperglycemic condition present dysregulation at receptor and intracellular signal transduction level, leading to hyperactive platelet adhesion, activation, aggregation, as well as thrombin activation [24]. Of note, platelets among patients with DM seem to be larger, more aggressive, and glycated. They express more glycoprotein IIb/IIIa surface receptors that mediate binding to von Willebrand factor, whereas they are more aggregated when stimulated by prothrombotic factors compared to patients without DM [25]. Similarly with endothelial cells, hyperglycemia triggers activation of PKC, leading to a decline in platelet-derived NO production accompanied by a rise in ROS secretion [26]. Furthermore, *in vivo* studies have highlighted intensified shear-induced platelet adhesion and aggregation, increased production of thromboxanes, and higher levels of circulating platelets in the activated state [27]. Adhesion molecules, such as P-selectin and E-selectin, are expressed by activated platelets and trigger platelet-leukocyte interplay, thus further exacerbating inflammation and thrombosis.

Impaired fibrinolysis is a key abnormality in DM and contributes to the adverse vascular outcomes in this population [28]. Plasminogen activator inhibitor-1 (PAI-1) is an important regulator of the fibrinolytic process and levels of this anti-fibrinolytic protein and tissue factor (TF) are elevated in atherosclerotic and non-atherosclerotic plaques of patients with DM, further deteriorating the hypercoagulable state [29]. Moreover, lower levels of thrombomodulin, antithrombin III, protein C and S, and prostacyclin, as well as higher levels of factors VII and VIII have been observed among patients with DM, leading to disturbances in the fibrinolytic process [30]. All these platelet abnormalities, in combination with the coagulopathy, will immediately trigger the formation of thrombus after plaque rupture and, in such manner, make the development of arterial occlusion more attainable.

Of note, autopsies and angiographic studies of patients with DM have illustrated that these patients more commonly have left main CAD, multivessel, or complex and diffuse disease involving the distal coronary tree [3]. Moreover, plaques rich in lipids, which are more susceptible to rupture, are also more frequent in patients with DM, thus predisposing them to ACS [26]. In more detail, patients with ACS and DM usually present with more fissured plaques and intracoronary thrombi, whereas coronary arteries in these patients are less likely to undergo favorable remodeling in response to atherosclerosis [3,31].

Consequently, there are plentiful pathophysiological components related to atherosclerosis, metabolic irregularities, and platelet abnormalities in DM that predispose to a poor prognosis combined with an unparalleled response to coronary revascularization following ACS. Therefore, patients with DM should be more aggressively treated when compared to the general population in terms of CV risk factors to protect from ACS.

## 3. Challenges in ACS Presentation and Diagnosis

Among patients presenting with ST-elevation myocardial infraction (STEMI), almost 25% of them have known DM and more than 40% shows prediabetes or previously undiagnosed T2DM along with a rapid increase in prevalence and mortality rates reported during recent decades [32,33]. However, ACS presentation and diagnosis among these patients remains challenging despite the achieved knowledge and awareness [34]. So far, it is well documented that patients with T2DM more often present late in the emergency department and with atypical symptoms or even silent myocardial ischemia when compared to those without DM, possibly due to the autonomous nervous system dysregulation, leading to delayed diagnosis and treatment [35,36]. Therefore, patients with T2DM often delay seeking medical attention when they experience symptoms, leading to worse outcomes in ACS. Moreover, they may not experience acute chest pain or chest discomfort directly associated with ACS but may present symptoms like shortness of breath, fatigue, nausea, or abdominal pain, which can be easily attributed to other diabetes-related complications or medical conditions that require further differential diagnosis.

Likewise, challenges in diagnosing ACS in this patient population derive from the fact that patients with T2DM more frequently suffer from multivessel disease and multiple coronary lesions, facing a higher percentage of vulnerable atherosclerotic plaques associated with impaired microvasculature vasodilation [37]. Interpreting electrocardiogram (ECG) findings and identifying new ischemic changes in these patients might be difficult, considering their potential ECG abnormalities at baseline [38]. Similarly, to identify yet another potential clinical challenge, biomarker interpretation might also be demanding, since increased levels of troponin can be also present in other cardiac and non-cardiac conditions [39]. Therefore, to address these challenges, healthcare professionals should maintain a high level of suspicion for ACS in patients with DM, conducting a thorough physical examination and appropriate testing to ensure timely diagnosis and treatment.

Finally, it is worth mentioning that in the emergency setting of acute chest pain, according to EPIC-ACS, an observational cohort study, the presence of epicardial adipose tissue (EAT) in patients was determined to be a strong and independent predictor of obstructive CAD and subsequently its assessment could enhance the diagnostic approach [40]. In this context, the meta-analysis upon which this study was based, provided proof of evidence that individuals with MI were characterized by the presence of more EAT when compared to their control counterparts [41]. In an attempt to clarify the mechanism accountable for this relationship, it was proposed that EAT functions as a large secretosome from which adipokines are excreted and exert their pro-inflammatory action on the myocardial cells. Furthermore, given the fact that a relationship between its volume and the extent of CVD and metabolic syndrome has been established, as well as a correlation between EAT volume and the onset and progression of CAD, this specific marker gained a greater clinical value. For that reason, quantification of this tissue has improved in recent years and even though imaging with cardiac magnetic resonance remains the “gold standard” method, echocardiography and cardiac computed tomography can be used as cost-effective and fast modalities to reveal EAT attenuation in the initial stages, which is associated with coronary artery calcification, AMI, and coronary adverse events [42,43].

## 4. Coronary Lesions in Patients with T2DM

Patients with DM are affected by multivessel CAD with a more diffuse coronary artery involvement than patients without DM, which may contribute to less favorable outcomes following revascularization [44]. Accordingly, early identification of high-risk patients for a CV event is of importance, while the increasing use of intracoronary imaging techniques in recent years provides a better understanding of plaque morphology.

CAD is the manifestation of atherosclerosis in the coronary vasculature defined by a greater than 50% diametral narrowing of an epicardial coronary vessel [45]. The pattern of involvement may be localized or diffuse. Frequently, endothelial dysfunction is present, regardless of the degree of manifestation [46]; even with angiographically localized findings, additional plaques are often present, appearing as wall irregularities on angiography [47]. Patients with DM and coronary atherosclerosis display a diffuse distribution pattern, with preferential involvement of the proximal coronary vessels and the main stem [45], as well as specific changes in the microcirculation with thickening of the basal membrane in the capillary region [48].

Moreover, chronic disturbances in the functional integrity of the vascular endothelium precedes the development of atherosclerosis. Endothelium-mediated vasodilation represents an important physiological adaptation mechanism in the presence of increased blood flow (stress, physical exertion). Vasodilation, which is predominantly mediated by prostacyclin, endothelium-derived relaxing factor (EDRF), and NO, is attenuated in patients with arterial hypertension, hypercholesterolemia, and DM [49]. This functional disorder of the endothelium is considered an early form of atherosclerosis. Meanwhile, mechanical stimuli (shear forces) as well as immunologic, inflammatory, and hemostasis-derived permeability disorders of the endothelium can cause mononuclear leukocytes to invade the intima and initiate the formation of arteriosclerotic plaques [50].

Concerning the characteristics of the coronary lesions in patients with T2DM, a study which analyzed the morphology of the plaques along with calcification using optical coherence tomography (OCT) prior to coronary intervention, showed that they had a lower minimal fibrous cap thickness and a higher percent area stenosis when compared to patients without DM. As a result, the minimal fibrous cap thickness overlying the necrotic lipid core, but not the calcification itself, is likely to contribute to the increased plaque vulnerability observed in patients with T2DM [51]. Another study, aiming also to investigate the coronary plaque phenotype of patients with T2DM at the setting of ACS using OCT, showed that the culprit lesions of this group appeared to have a higher prevalence of lipid-rich plaque and macrophage accumulation when compared to the non-DM group. Therefore, in relation to the non-culprit lesions, patients with T2DM exhibited a greater maximal lipid arc, thinner fibrous cap thickness, and a higher prevalence of thin cap fibroatheroma. Thus, patients with T2DM develop more vulnerable features in both culprit and non-culprit lesions, indicating a higher level of vascular instability [52].

## 5. Revascularization Strategies

Patients with DM present complex coronary lesion morphology, including involvement of multiple vessels and possibly left main stem involvement. Accordingly, the choice of coronary revascularization in symptomatic patients is notably meaningful, particularly in patients with non–ST-segment elevation ACS (NSTE-ACS). Considering that most patients with T2DM have multiple coronary artery involvement, the choice of action should be based on an interdisciplinary decision, since most patients with T2DM may benefit from an aortocoronary bypass, especially in the long-term, as evidenced by a reduction in all-cause mortality and a lower rate of myocardial infraction (MI) [53]. Indeed, based on the available studies, CABG may improve long-term outcomes after an ACS with T2DM [54].

Therefore, in NSTE-ACS, decisions on the management of revascularization are more challenging. According to most guidelines, a coronary catheterization should be performed within 24–72 h after the onset of the NSTE-ACS [55]. CABG might also be the favorable revascularization method for patients with T2DM and multivessel CAD in the NSTE-ACS setting. However, there is a lack of randomized clinical trials [56]. In patients with two-vessel CAD without a proximal left anterior descending lesion, only a relatively small percentage of them, namely 15%, received CABG. When percutaneous coronary intervention (PCI) with bare metal stents, and first-generation drug-eluting stents was compared to CABG in T2DM patients with multivessel CAD, similar mortality rates were found in those undergoing CABG [57]. On the other hand, almost 60% of patients with triple-vessel disease with proximal left anterior descending artery involvement will be treated surgically. In patients who present with an STE-ACS, the instant revascularization of the culprit lesion with PCI is superior to CABG or fibrinolysis, while this decision is often not influenced by the presence of DM [55]. Still, the preferred revascularization strategy of residual multivessel CAD after primary PCI for STE-ACS patients remains a controversy and should be an issue of further study. Controversially, current guidelines still tend to advocate CABG over PCI for revascularization in this group of patients as a reflection of previous study results comparing PCI with bare-metal stents to CABG, before drug-eluting stents and glycoprotein IIa/IIIB inhibitors became standard of care [57]. Regarding the comparison of PCI with second-generation drug-eluting stents to CABG, studies and retrospective analyses have not demonstrated a statistically significant benefit favoring one or the other with regard to mortality rates [58]. Therefore, the use of second-generation drug-eluting stents, such as zotarolimus-eluting and everolimus-eluting stents, is recommended. The decision on the best revascularization strategy can be additionally based on the SYNTAX score, which allows the validation of the morphologic extent of the CAD. PCI would be an appropriate alternative in patients with diabetes and a less complex coronary artery disease with a lower SYNTAX score. Whereas in cases of a complex anatomy of both the stenosis, as well as the coronary vessels, CABG would be more suitable [59].

Whether it is useful to determine fractional flow reserve (FFR) for treatment decisions was investigated by the PRIME-FFR study [60]. Patients were recruited from other studies in which the therapeutic strategy had initially been planned based on angiography before measurement of FFR. After FFR determination, reclassification occurred, and the final strategy was determined. Regardless of the outcome of FFR and the impact on treatment decisions, patients with DM had a higher rate of major adverse CV events (MACE), a higher rate of infarction, and a higher all-cause mortality (5.3 vs. 3.6%). There was no statistical difference in the one-year rate of MACE in patients with reclassification vs. without reclassification. However, regardless of T2DM, the MACE rate was highest (6.6%) in patients in whom the FFR result was ignored in the treatment decision. The measurement of arterial flow reserve is particularly justified in patients with DM and often leads to a change in the originally planned therapy. Even then, however, the prognosis in patients with DM is less favorable than in patients without normal glycose metabolism [60].

## 6. Antithrombotic Therapy

In patients with ACS, initiation of antithrombotic therapy should be as early as possible. While the initial administration of acetylsalicylic acid is recommended for all patients with ACS, provided there are no contraindications, the second antiplatelet agent should be selected individually according to the overall clinical presentation [55].

According to the current ESC guidelines, there is no difference in the recommended antithrombotic therapy in patients with ACS and DM or not [61]. Nevertheless, several studies show an increased thrombotic risk in patients with DM. Several mechanisms for increased platelet activity in patients with DM have been demonstrated [62]. Platelets express insulin receptors on their surface that inhibit platelet activity via calcium influx control. When insulin resistance exists, the calcium influx is impaired, resulting in increased platelet activity.

Furthermore, impaired production of the vasodilator nitric oxide was found in platelets from patients with DM who also highly express the potent vasoconstrictor thromboxane A2 [63]. Also, increased expression of P-selectin and glycoprotein IIb/IIIa receptor (GPIIb/IIIa) on platelets leads to a prothrombotic state. In patients with DM, different doses of acetylsalicylic acid [64] or clopidogrel have been tested [65]. Significant reductions in platelet activity and ischemic events were observed for higher doses, but with a concomitant increased bleeding risk, therefore higher doses are not currently recommended by guidelines [55].

Despite the increased expression of GPIIb/IIIa receptors on platelets in patients with DM, the routine use of GPIIb/IIIa receptor antagonists in the setting of ACS is not recommended. Although a survival benefit was demonstrated in patients with NSTE-ACS and DM with early administration of GPIIb/IIIa receptor antagonists in a meta-analysis [66], this finding was not confirmed in the EARLY-ACS trial [67].

Prasugrel and ticagrelor achieve efficient platelet function inhibition. Both a sub-study of the TRITON-TIMI-38 trial for prasugrel [68] and a sub-study of the PLATO trial for ticagrelor [69] showed the best response in terms of a reduction in stent thrombosis rates and the combined endpoint (CV death, myocardial infarction, and insult) in patients with DM [61].

## 7. Glucose Management in the Acute Phase of ACS

A U-shape pattern between glucose levels and mortality exists in patients with DM and ACS. Both hyperglycemia and hypoglycemia are related to similar adverse effects on in-hospital and 6-month mortality [70]. Hyperglycemia is associated with impaired microvascular function so, when persistent, it is related to increased mortality risk. Hypoglycemia also, in patients with DM and ACS, unveils worse outcomes. Therefore, moderate control of the blood glucose levels in these patients was strongly highlighted by the landmark of the Normoglycemia in Intensive Care Evaluation-Survival Using Glucose Algorithm Regulation (NICE-SUGAR) trial, which concluded blood glucose goals at <180 mg/dL while avoiding hypoglycemia [71].

Even though it is yet to be clarified whether high blood glucose levels in patients with DM are a direct mediator of increased mortality and complications in the setting of ACS, or merely a marker indicating an amplified severity of the disease and comorbidity. The Diabetes Mellitus, Insulin Glucose Infusion in Acute Myocardial Infarction (DIGAMI) study randomized subjects with DM displaying symptoms within the first 24 h of acute MI (AMI) onset or baseline glucose levels > 198 mg/dL. The two groups that were formed were the intervention arm, which received intravenous dextrose-insulin infusion for ≥24 h, a scheme that was replaced by subcutaneous insulin injections three times daily, later succeeded by a long-term subcutaneous insulin-based control of the blood glucose levels with the goal of sustaining them at 126–180 mg/dL, and the control arm that received standard of care. Data analysis from this study revealed a significant mortality difference in the intervention group at 1 and 3 years but not at 3 months or throughout the hospitalization despite the differences in the blood glucose levels achieved between the studied groups [72,73]. These results urged the broader Diabetes Mellitus, Insulin Glucose Infusion in Acute Myocardial Infarction DIGAMI-2 trial to investigate whether the survival benefits presented in the DIGAMI study were attributed to short-term or long-term glucose lowering with insulin in patients with AMI, but due to limitations could not provide a definitive answer [74]. Beyond these studies, the HI-5 study included ACS patients with or without DM but with an initial glucose ≥ 140 mg/dL at admission and randomized them to an IV insulin drip with a target blood glucose level of 72–180 mg/dL versus standard of care and managed to confirm a statistically significant mortality benefit in the intervention arm between hospitalization and 6 months [75].

Further studies, such as the Low-Dose Glucose-Insulin-Potassium is Ineffective in Acute Myocardial Infarction: Results of a Randomized Multicenter Pol-GIK Trial [76], the Groningen Intervention Study for the Preservation of Cardiac Function GIPS [77], and the Effect of Glucose-Insulin-Potassium Infusion on Mortality in Patients With Acute ST-Segment Elevation Myocardial Infarction: The CREATE-ECLA Randomized Controlled Trial [78], were aimed at assessing the glucose-insulin-potassium (GIK) therapy without the need of a target-driven glucose level in the setting of ACS. In this way, the GIK studies cannot lead to decisions about glucose management in this group of patients [79]. With reference to the other anti-diabetic agents, even though there is some available data investigating their effect on cardiovascular prognosis, their possible implementation in the acute setting has not yet been explored. Nothing but a single small prospective observational study, which tested the hypothesis of whether sodium-glucose cotransporter 2 inhibitors (SGLT-2i) could improve left ventricular (LV) diastolic function following an ACS, is available with respect to this topic,. The trial design was based on the initiation of empagliflozin therapy at discharge in patients with T2DM after an ACS and concluded that the addition of this agent was indeed associated with a reduction in LV mass along with favorable changes in diastolic function parameters [80,81,82].

## 8. Newer Antidiabetic Agents for the Post-Acute Phase of ACS

Several classes of the new generation of antidiabetic agents have so far been used in the ACS setting, but current data does not allow any definitive conclusions, as is further discussed (Table 1).

### 8.1. Sodium Glucose Transporter 2 Inhibitors and Acute Myocardial Infarction

SGLT2i reduced the risk of CV death in patients with T2DM associated with a mean 23% relative risk reduction in hospitalizations attributed to HF with or without the presence of an existing history of HF [89,90,91]. Furthermore, the DAPA-HF and EMPEROR-Reduced trials, concerning patients with established stable chronic HF with reduced ejection fraction (HFrEF), indicated that the addition of SGLT2i in the subjects’ medication reduced the composite of total CV deaths or hospitalizations for HF [92,93]. Moreover, the EMPA-RESPONSE-AHF, a randomized, double-blind, placebo-controlled, multicenter pilot study on the influence of empagliflozin on clinical outcomes in patients with acute decompensated HF, the EMPULSE which investigated the impact of empagliflozin in patients hospitalized for Acute HF, and the SOLOIST-WHF, that investigated the effects of Sotagliflozin in patients with DM and a history of HF that recently worsened, showcased that initiating guideline-directed medical therapies with SGLT2i in patients with acute HF during their hospitalization or shortly thereafter (within 3 days of discharge) is safe and, more importantly, significantly minimizes the risk of rehospitalization after discharge [94,95]. However, all these trials excluded individuals with a recent history of MI within the past three months, so it remains undecided whether these agents are effective and harmless to use early post-MI.

There is still narrow clinical evidence on the use of gliflozins in ACS and most of them derive from experimental models. Administration of canagliflozin in male rat models in the acute setting of ischemia reduced myocardial infarct size and enhanced left ventricular systolic and diastolic function [96]. Similar results were obtained with dapagliflozin [97] and empagliflozin [98].

Recently, the Empagliflozin in AMI (EMMY) trial investigated the use of empagliflozin within 72 h after PCI and provided evidence of improvement in echocardiographic functional and structural parameters, in addition with a significantly greater NT-proBNP reduction [86]. In addition, according to the EMBODY trial that randomized subjects with AMI and T2DM and investigated the action of empagliflozin on cardiac sympathetic activity, the group of patients who received empagliflozin was distinguished from the control group by a safe and effective reduction of the incidence of arrhythmias and prevention of the adverse implications of myocardial ischemia on kidney function [85]. The SGLT2-I AMI PROTECT Registry, showed lower rates of atrial fibrillation and VT/VF episodes, as well as lower rates of in-hospital cardiovascular death and HF hospitalization [88]. A single-center retrospective study which enrolled 786 patients with AMI provided evidence that the administration of dapagliflozin was associated with reduced MACE in these patients, and especially those described as seniors and hypertensive, including those who did not receive ARNI [87]. Presently, there are two ongoing trials testing the efficacy and safety of SGLT2 inhibition in patients with AMI, the EMPACT-MI (Trial to Evaluate the Effect of Empagliflozin on Hospitalization for Heart Failure and Mortality in Patients With Acute Myocardial Infarction) for empagliflozin and the DAPA-MI (Dapagliflozin Effects on Cardiovascular Events in Patients With an Acute Heart Attack) for dapagliflozin [82,99], that are anticipated in order to further clarify this issue, especially given the fact that data from an observational prospective study have established a positive impact of the use of SGLT2i on both structural and functional cardiac parameters in diabetic subjects with a preserved EF [100].

### 8.2. Mechanism of SGLT2i Beneficial Effects

**Figure 2:**
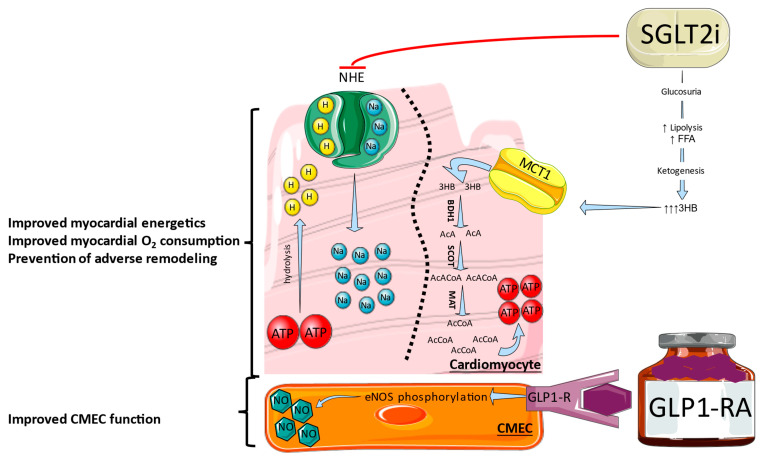
Mechanisms of cardiac protection of SGLT2i and GLP1-RA. SGLT2i, by inhibiting the sodium-hydrogen exchanger (NHE) and improving myocardial energy fuel metabolism towards ketones, promote an enhanced myocardial oxygen consumption and prevent adverse remodeling. GLP1-RA lead to endothelial nitric oxide synthase (eNOS) phosphorylation and increased NO bioavailability in coronary microvascular endothelial cells, improving their function. 3HB: 3-hydroxybutyrate, AcA: acetoacetate, APT: adenosine triphosphate, BDH1: 3HB dehydrogenase-1, CMEC: coronary microvascular endothelial cell, FFA: free fatty acids, MAT: 2-methylacetoacetyl-coenzyme A thiolase, MCT1: monocarboxylate transporter-1, SCOT: succinyl-CoA:3-oxoacid-CoA transferase, ↑: Increase, ↑↑↑: highly increase.

In circumstances of normal oxygenation, myocardial metabolism employs mainly fatty acids (60%) and glucose (30%), whereas in hypoxic conditions like myocardial infarction there is a shift to anaerobic metabolism and ketones become the predominant energy substrate. One hypothesis is that SGLT2i improves myocardial fuel metabolism, contractility, and cardiac efficiency by causing an alteration in energy substrate use from lipids and glucose to ketone bodies (Figure 2) [101]. Empagliflozin leads to an increase in circulating levels of ketones, as well as the myocardial expression of the ketone body transporter, which is linked with an upsurge of cardiac ATP production in rats undergoing permanent coronary artery ligation [102]. Although empagliflozin did not reduce the infract size, presumably due to the absence of reperfusion, the increased circulating ketone levels might be a probable mechanism of its cardioprotective action on cardiac remodeling, conceivably due to the upregulation of crucial oxidative phosphorylation mediators, leading diminished oxidative stress [103]. Moreover, aside from the decreased blood glucose levels, SGLT2i boosted 3- hydroxybutyrate (3HB) levels implying the utilization of ketone bodies. More specifically, elevated consumption of 3HB in animal models is linked to an improvement in cardiac function, cardiac remodeling in pathologic conditions, and oxygen uptake [104]. Since the use of ketones relies on the targeted organ, the heart as well as the kidneys seem to be the ones mostly favored by an increase in 3HB [105].

Another potential protective mechanism of the SGLT2i is the inhibition of Na^+^-H^+^ exchanger (NHE). The intracellular accumulation of Na^+^ and Ca^2+^ has a crucial function in ischemia-induced myocardial injury. During ischemia, ATP hydrolysis produces H^+^ and the accumulation of intracellular H^+^ and, together with the activation of Na^+^-dependent pH regulatory mechanisms, contributes to Na^+^ accumulation. Intracellular Na^+^ accumulation, coupled with the NHE, then causes Ca^2+^ overload and further LV mechanical dysfunction [106]. Even if reperfusion takes place, there can be reperfusion injury called the pH paradox, in which reperfusion restores pH by rapid extraction of accumulated H^+^, activation of the NHE, and, consequently, a precipitous accumulation of Na^+^ that could result in cellular oedema and calcium overload [107]. SGLT2i not only regulate the mechanical function of the myocytes by modifying Ca^2+^ cycling but, at the same time, provide myocardial protection through the maintenance of suitable myocardial redox balance. In addition, inhibition of NHE lessens the cardiomyocyte injury, remodeling, and systolic dysfunction [108]. The actions induced on NHE by empagliflozin have been verified in murine cardiomyocytes and in the *ex vivo* intact heart, where this agent produced direct cardiac effects by inhibiting NHE at the setting of ischemia [109]. Also, *ex vivo* administration of canagliflozin adequately suppressed the activity of NHE [110], while canagliflozin seems to preserve cardiac contractility during myocardial ischemia in the *in vivo* setting. Nonetheless, in relation to diabetic and non-diabetic hearts, when canagliflozin was administrated *ex vivo*, it did not attenuate infarct size, leading to the assumption that NHE inhibition is not the exclusive mechanism through which protection against acute ischemia/reperfusion injury can be achieved [111]. In human atrial and ventricular myocardium of patients with HF, empagliflozin inhibits NHE in cardiomyocytes and the inhibition was similar in mouse ventricular myocytes [112]. Finally, empagliflozin was found to reduce NHE and oxidative stress in human coronary endothelial cells supporting the initial hypothesis [113].

SGLT2i have been found to reduce the risk of ventricular arrhythmias. This finding is clinically relevant since ventricular arrhythmias are jointly related to sudden cardiac death in the initial stages of AMI [114]. In a post hoc analysis of the DAPA-HF trial, dapagliflozin was found to reduce the risk of ventricular arrhythmias or sudden cardiac death when administered on top of the standard of care in individuals HFrEF [115]. Another meta-analysis has highlighted the protective effect of SGLT2i against ventricular arrhythmias, irrespective of comorbidities or baseline conditions [116]. Nevertheless, none of these mechanisms are specifically investigated in patients with AMI. In rat models, pretreatment with empagliflozin diminished myocardial vulnerability to sudden cardiac death and decreased the susceptibility to reperfusion-induced arrhythmias [117]. Likewise, in ischemia/reperfusion rabbit models, empagliflozin was shown to lower the incidence of ventricular arrhythmias [118]. In ventricular cardiomyocytes isolated from rats, dapagliflozin attenuated the current Ik accountable for the probable QT prolongation, reducing the risk of cardiac arrhythmias [119]. Recently, the EMBODY trial, exploring the effects of SGLT2i on cardiac sympathetic and parasympathetic activities in subjects with T2DM suffering from an AMI, demonstrated that the group receiving empagliflozin exhibited a considerable improvement in both cardiac sympathetic and parasympathetic nerve activities when compared to the control group [85]. SGLT2i modulate sympathetic tone and this has been proposed as a potential mechanism of cardiac protection, as patients on SGLT2i therapy had more balanced autonomic system activity in comparison with non-users [120]. Still, there is a need for more data on the antiarrhythmic effects of SGLT2i, even though there are assumptions regarding a possible relationship with these outcomes through the reduction of the inflammatory burden, admission stress hyperglycemia, LV infarct size, and calcium-related arrhythmogenesis.

### 8.3. Glucagon-like Peptide-1 Receptor Agonists and Dipeptidyl-Peptidase-4 Inhibitors in Acute Coronary Syndrome

Glucagon-like peptide-1 receptor agonist (GLP1-RA)-based therapies could be useful for glucose control since these agents rarely lead to hypoglycemia and, additionally, do not need monitoring of glucose levels or dose adjustment. Also, GLP1 besides glucose control exerts encouraging effects on the cardiovascular system. In cardiomyocytes isolated from GLP1 receptor knockout mice, administration of lixisenatide in the acute setting reduced infarct size and improved cardiac function [121]. Furthermore, albiglutide reduced myocardial infarct size, improved cardiac function, and was correlated with an augmented glucose uptake from the myocardial cells and a shift toward a more energetically favorable substrate metabolism when administered in rat models [122]. Similar results were found with lixisenatide [120]. Diabetic mice pretreated with liraglutide before the induction of MI showed better survival, reduced infract size, and improved cardiac output [123]. GLP1 can exert a protective effect against endothelial dysfunction [124] and Exendin-4 could augment eNOS phosphorylation and nitric oxide (NO) production, pathways that are associated with vascular relaxation [125]. Also, chronic treatment with GLP1-RA has been shown to reduce blood pressure and plasma levels of apo-lipoprotein B, thus lowering coronary atherosclerotic burden and inducing a better hemodynamic profile in the setting of AMI [126,127].

In 10 patients with AMI and left ventricular dysfunction, a 72 h infusion of GLP1 (7–36) amide after primary PCI generated an improvement in global and regional LV wall motion scores and reduced hospital stay [128]. In a prospective pilot study, exenatide was administered in 40 patients that were admitted to a cardiac intensive care unit and there was no difference in performance in the attainment of a target glucose range of 5.6 to 7.8 mmol/L and control was achieved without episodes of severe hypoglycemia, but nausea was a frequent adverse event [129]. In a larger RCT of 172 patients with STEMI, exenatide was assigned intravenously 15 min before PCI and maintained for 2 h after the intervention, leading to increased myocardial salvage and decreased final infarct size by almost 30% [130,131]. Similar results were found in other small trials with STEMI patients who received exenatide or liraglutide [132,133]. Liraglutide was evaluated in 90 patients with NSTEMI, resulting in improved LV ejection fraction by 4.7% compared to placebo after 3 months, together with reduced in inflammation and oxidative stress [134].

DDP-4 is an enzyme of which the enzymatic activity can be measured in plasma and can be also detected in the endothelium. It degrades incretins GLP1 and GIP and, when the activity of the enzyme is blocked thought DPP-4 inhibitors, glycose homeostasis is improved [135]. The Examination of Cardiovascular Outcomes with Alogliptin versus Standard of Care (EXAMINE) trial, in subjects with DM and a recent ACS within 15 to 90 days before recruitment, proved the safety of DPP-4 inhibitors compared to placebo, with no increase in the rates of major adverse cardiovascular events [83]. Additionally, the Evaluation of Lixisenatide in Acute coronary syndrome (ELIXA) trial, in which 6068 patients were randomized within 180 days of an ACS that required hospitalization, demonstrated that lixisenatide was non-inferior to placebo for the composite of cardiovascular death, non-fatal MI, non-fatal stroke, hospitalization for unstable angina, or heart failure [84]. Moreover, treatment with sitagliptin in patients with ACS and DM did not increase natriuretic peptide levels post-AMI, without altering platelet aggregation [136]. Additionally, post-ACS sitagliptin did not improve endothelial function [137]. In another study post-ACS with intravascular imaging (ESPECIAL-ACS), it was found that although sitagliptin treatment for 6 months did not affect coronary plaque volume, it reduced the lipid plaque volume towards a more stabilized phenotype [138]. However, controversy exists on the impact of saxagliptin and alogliptin in patients with HF [139,140]. Taken together, treatment with most DPP4-inhibitors in patients with DM is safe, with a neutral balance regarding cardiovascular benefits.

## 9. Conclusions

DM is found in a significant proportion of patients with ACS and is associated with adverse short- and long-term outcomes. Serum glucose control is essential even in the acute phase to reduce complications, and both hyperglycemia and hypoglycemia should be avoided. However, the best treatment strategy is yet undefined. Non-insulin therapies, due to the low risk of hypoglycemia concurrently with the multifactorial CV protective effects, may end up as the best treatment option in the future. Nevertheless, little evidence exists in the setting of ACS for GLP1-RA, DPP-4- inhibitors and SGLT2i.

## Figures and Tables

**Table 1:** New antidiabetic medications in the setting of acute coronary syndromes.

Clinical Trial, Year	Ref.	Medication	Study Type	Study Population	Intervention	Main Findings
EXAMINE, 2013	[83]	Alogliptin	Double-blind, noninferiority randomized trial	Patients with T2DM and AMI or UA requiring hospitalization within previous 15–90 days	Alogliptin or placebo added to existing antidiabetic and CV therapy	Composite primary endpoint of CV death, nonfatal-MI and -stroke was 11.3% in the alogliptin group and 11.8% in the placebo (*p* < 0.001 in noninferiority)
ELIXA, 2015	[84]	Lixisenatide	Multicenter, double-blind, randomized	Patients with T2DM and AMI or UA within the previous 180 days	Lixisenatide or placebo added to locally determined standard of care	Non-inferiority but also non-superiority of lixisenatide to placebo for the primary endpoint of MI, CV death, stroke, hospitalization for UA; similar rates of SAE
EMBODY, 2020	[85]	Empagliflozin	Prospective, multicenter, double-blind, randomized	Patients with AMI and T2DM	Addition of either EMPA 10 mg or matching placebo	SDANN changes both in the EMPA (*p* = 0.02) and placebo groups (*p* = 0.06); LF/HF ratio altered in both EMPA (*p* = 0.01) and placebo groups (*p* = 0.43); improvement in HRT only in the EMPA group
EMMY, 2022	[86]	Empagliflozin	Multicenter, double-blind, randomized	Patients with AMI and elevation of CK > 800 IU/L undergoing PCI	Addition of either EMPA 10 mg or matching placebo once daily within 72 h of PCI	Fifteen percent lower levels of NT-proBNP; 1.5% absolute improvement of LVEF; 6.8% greater reduction of mean E/e’; no change in adverse events
Zhu et al. 2022	[87]	Dapagliflozin	Single-center, retrospective	Patients with AMI	Dapagliflozin or not at discharge	The cumulative incidence of MACE (log-rank test, *p* = 0.009), HF (*p* = 0.003), nonfatal MI (*p* = 0.005), and URR (*p* = 0.031) was higher in the DAPA-free group
SGLT2-I AMI PROTECT Registry, 2023	[88]	SGLT2i	Multicenter international registry	Patients with AMI undergoing PCI between 2018 and 2021	SGLT2i versus non-SGLT2i users	SGLT2-I users had lower rates of in-hospital CV death, arrhythmic burden, and CI-AKI (all *p* < 0.05); lower HF hospitalization and CV death during median follow-up of 24 ± 13 months (*p* < 0.04 for all)

AMI: acute myocardial infarction; NT-proBNP: N-terminal pro-hormone of brain natriuretic peptide; SDANN: standard deviation of all 5 min mean normal RR intervals; EMPA: empagliflozin; LF/HF: the low-frequency-to-high-frequency ratio; HRT: heart rate turbulence; HF: heart failure; CI-AKI: contrast-induced acute kidney injury; CV: cardiovascular; MI: myocardial infarction; PCI: percutaneous coronary intervention; MACE: major adverse cardiovascular events; URR; unplanned repeat revascularization; UA: unstable angina; SAE: serious adverse events; SGLT2i: Sodium-glucose cotransporter-2 inhibitors; DAPA: dapaglifozin.
